# Gut Microbiota and Type 2 Diabetes Mellitus: Association, Mechanism, and Translational Applications

**DOI:** 10.1155/2021/5110276

**Published:** 2021-08-17

**Authors:** Lili Zhang, Jinjin Chu, Wenhao Hao, Jiaojiao Zhang, Haibo Li, Chunjuan Yang, Jinghan Yang, Xiaohua Chen, Honggang Wang

**Affiliations:** ^1^Central Laboratory, Weifang People's Hospital, Weifang 261000, China; ^2^Department of Scientific Research Management, Weifang People's Hospital, Weifang 261000, China; ^3^Central Laboratory of the First Affiliated Hospital, Weifang Medical University, Weifang 261000, China; ^4^Department of Nuclear Medicine, Weifang People's Hospital, Weifang 261000, China; ^5^Clinical Laboratory, Weifang People's Hospital, Weifang 261000, China

## Abstract

Gut microbiota has attracted widespread attention due to its crucial role in disease pathophysiology, including type 2 diabetes mellitus (T2DM). Metabolites and bacterial components of gut microbiota affect the initiation and progression of T2DM by regulating inflammation, immunity, and metabolism. Short-chain fatty acids, secondary bile acid, imidazole propionate, branched-chain amino acids, and lipopolysaccharide are the main molecules related to T2DM. Many studies have investigated the role of gut microbiota in T2DM, particularly those butyrate-producing bacteria. Increasing evidence has demonstrated that fecal microbiota transplantation and probiotic capsules are useful strategies in preventing diabetes. In this review, we aim to elucidate the complex association between gut microbiota and T2DM inflammation, metabolism, and immune disorders, the underlying mechanisms, and translational applications of gut microbiota. This review will provide novel insight into developing individualized therapy for T2DM patients based on gut microbiota immunometabolism.

## 1. Introduction

Diabetes mellitus (DM) is a group of chronic metabolic diseases characterized by hyperglycemia. There are two most common forms, namely, type 1 diabetes mellitus (T1DM) and type 2 diabetes mellitus (T2DM). T2DM accounts for about 90% of all diabetes cases, resulting from insulin resistance combined with impaired insulin secretion. DM can cause a variety of acute and chronic complications, such as blindness, amputation, heart disease, kidney failure, and premature death. According to the latest version of the diabetes map released by the International Diabetes Federation (IDF) in 2019, about 463 million adults worldwide suffer from diabetes, with an average growth rate of 51%. The number of diabetic patients will reach 700 million by 2045 with increasing economic burden for diabetes worldwide [1]. The pathogenesis of diabetes is complex and unclear. Accumulated evidence has implicated genetics, infection, immune disorders, obesity, and diet are closely related to diabetes. Diet control, reasonable exercise, oral antidiabetic drugs, and insulin injection are routine options for the prevention and treatment of diabetes. However, none of them can fundamentally prevent the development of diabetes and associated complications. During the last decade, the role of gut microbiome has drawn much attention across the world. Understanding the interplay of gut microbiome and diabetes would provide new insight into developing therapeutics for diabetes.

With the development of high-throughput sequence, research on gut microbiota breaks through the shackles of the traditional manner of living bacterium cultivation. The whole picture of gut microbiota is gradually revealed. Gut microbiota consists of more than 1000 bacterial species, mainly distributing in 9 phyla, most of which belong to the *Firmicutes*, *Bacteroidetes*, *Proteobacteria*, and *Actinobacteria* [2]. The main physiological functions of gut microbiota include the following: food digestion and absorption, enhanced host immune, biological antagonism, strengthened antitumor responses, and synthesized beneficial compounds [3, 4]. Once the gut microbiota is out of balance, a series of diseases would be induced, including metabolic diseases, cardiovascular and cerebrovascular diseases, autoimmune diseases, inflammatory bowel disease, psychotic disorders, and cancer [5].

A number of studies have demonstrated that gut microbiota plays an important role in T2DM. Gut microbiota participates in regulating glucose and insulin sensitivity. Symptoms of diabetic patients can be improved by modifying gut microbiota, which helps to reverse the impaired glucose tolerance and fasting glucose in prediabetes. This review will focus on elucidating the correlation between gut microbiota and T2DM, the pathogenesis of T2DM mediated by gut microbiota, and the therapeutic interventions based on gut microbiota.

## 2. Gut Microbiota of T2DM

The first study on gut microbiota of T2DM was reported in 2010 [6]. It has been found that the abundance of class *Clostridia* and phylum *Firmicutes* in T2DM patients considerably declined, while the level of class *Betaproteobacteria* was highly increased and positively associated with plasma glucose. Additionally, the ratios of *Bacteroidetes* to *Firmicutes* and *Bacteroides*-*Prevotella* to *C. coccoides*-*E. rectale* are positive correction with plasma glucose, suggesting that T2DM is correlated with the intestinal microbiota composition. In 2012, a metagenome-wide association study (MGWAS) has revealed the characterization of gut microbiota in T2DM [7]. Herein, moderate gut microbial dysbiosis is applied to characterize the gut microbiota of T2DM patients. The abundance of butyrate-producing bacteria (*Clostridiales* sp. SS3/4, *Faecalibacterium prausnitzii*, *Roseburia intestinalis*, *Eubacteriumrectale*, and *Roseburia inulinivorans*) is decreased, while the abundance of opportunistic pathogen bacteria (*Bacteroides caccae*, *Clostridium hathewayi*, *Clostridium symbiosum*, *Eggerthella*, *lenta Clostridium ramosum*, and *Escherichia coli*), mucin-degrading bacteria (*Akkermansia muciniphila*), and sulfate-reducing bacteria (*Desulfovibrio* sp. 3_1_syn3) is increased. A gut-microbiota-based T2DM classifier system can accurately classify T2D individuals, and the area under the receiver operating characteristic (ROC) curve is 0.81. Functional analysis has shown that glucose membrane transport, methane metabolism, heterogeneous biomass degradation, branched-chain amino acid transport and metabolism, and sulfate reduction pathways are enriched in patients with T2DM. Some functional genes related to flagella assembly, bacterial chemotaxis, butyrate biosynthesis, cofactors, and vitamin metabolism are decreased, while the activity of seven antioxidative stress-related enzymes is upregulated in T2DM [7]. A previous study has suggested that four intestinal *Lactobacillus* species increased in patients with diabetes, while the abundance of five *Clostridium* species decreased [8]. *Lactobacillus* is positively correlated with fasting blood glucose (FBG) and glycosylated hemoglobin (HbA1c), while *Clostridium* is negatively associated with HbA1c, FBG, C peptide, insulin, and plasma triglyceride and positively correlated with high-density lipoprotein (HDL) and adiponectin [8]. The metagenomic-cluster-based T2DM identification model showed superior discriminatory power (AUC = 0.83), in which *Roseburia* and *Faecalibacterium prausnitzii* displayed highly discriminant for T2DM. Regarding the gut barrier function, signaling pathways regarding energy metabolism and absorption, glycerides, and fatty acid synthesis, cysteine and methionine metabolism are also activated [8]. Taken together, different cohort studies have shown inconsistent findings. It has also been found that the differences were mainly associated with metformin treatment, rather than diabetes itself. Excluding the biological interference of metformin, the reduction of *Roseburia* spp., *Subdoligranulum* spp., and *Clostridiales* spp. was significantly correlated with T2DM, and the trend of enriched *Lactobacillus* was reversed [9]. In addition, metformin treatment leads to higher abundance of *Escheria* and lower abundance of *Intestinibacter*, which can be explained by the adverse gastrointestinal reactions such as diarrhea, nausea, vomiting, and abdominal distension [9]. Further analysis based on gut microbial function showed that metformin treatment reduced the intestinal lipid absorption and LPS-induced inflammatory response and increased the production of butyrate and propionate [9]. In addition, many studies have further confirmed the importance of metformin in regulating gut microbiota [10, 11]. It is suggested that the study of the correlation between gut microbiota and diabetes has been proved as an alarm for the impact of antidiabetic agents on gut microbiota homeostasis with T2DM; therefore, any treatment on gut microbiota should be carefully applied.

During the past few years, researchers have investigated the role of gut microbiota in prediabetes patients or newly diagnosed without antidiabetic drugs, hoping to better explain the correlation between diabetes and gut microbiota. Allin et al. have found that the level of *Clostridium* and *Akkermansia muciniphila* decreased significantly, while the level of *Dorea*, *Ruminococcus*, *Sutterella*, and *Streptococcus* increased, implicating that abnormal changes in gut microbiota occurred in the period of prediabetes [12]. Another study performed in the Swedish population has demonstrated that compared with the normal glucose tolerance (NGT) group, the composition of intestinal flora in impaired glucose tolerance (IGT), impaired fasting glucose and glucose tolerance (CGT), and untreated diabetes (T2D) groups has significantly changed, but no significant change was observed in the impaired fasting glucose (IFG) group [13]. It could be concluded that gut microbiota plays an important role in leading to diabetes by regulating systemic insulin resistance. Besides, in cases of prediabetes and T2D patients, the abundance of several butyrate producers decreased, such as *Pseudoflavonifractor* spp., *Clostridium* spp., *Alistipes* spp., *Faecalibacterium* spp., and *Oscillibacter* spp. The AUC of this gut-microbiota-based T2DM classifier model was 0.7. Therefore, its prediction power in distinguishing individuals with T2DM from those with NGT was moderate. However, an improved model for distinguishing T2DM and CGI had better AUC (AUC = 0.78), implicating the gut-microbiota-based classifier system could accurately assess the blood glucose status. Moreover, the ability of certain gut microbiota producing butyrate was inhibited, while genes involved in the biosynthetic pathway of intestinal biotin were significantly upregulated [13]. Accordingly, gut microbial dysbiosis plays a critical role in the pathogenesis of diabetes, which can change IGT rather than IFG.

In summary, the gut microbiota has been considered as a marker of metabolic diseases including T2DM [14]. Butyrate, metabolites of intestinal bacteria, can affect the insulin sensitivity. A decrease of butyrate has been proved to be positively correlated with diabetes [15]. Therefore, increasing the abundance of butyrate-producing bacteria or improving the butyrate synthesis ability of intestinal bacteria may be an effective method to prevent or treat diabetes.

## 3. Causality between Gut Microbiota and T2DM

Gut microbiota studies in large cohorts have emphasized the complex association between intestinal flora and T2DM. Animal studies from sterile mice have determined that gut microbiota is the vital cause of disease [16]. The sterile mice have lower insulin resistance and lower level of body fat than conventional mice. Subsequent gut microbiota transplantation tests have also confirmed that obesity and insulin resistance are significantly increased in sterile mice treated with ob/ob mice gut microbiota [17]. However, the results presented in sterile mice could not be used as a direct proof for human research due to the different genetic backgrounds [18]. A human study from 952 volunteers in the Netherlands finally knocked down the real hammer for the causal relationship between gut microbiota and T2DM [15]. Researchers have analyzed the whole genome and intestinal metagenome of subjects, measured fecal short-chain fatty acid (SCFA) levels, and counted clinical parameters. The causality between microbial characteristics and blood glucose characteristics was evaluated by bidirectional Mendelian Randomization (MR) analyses. It has been found that the increase of butyrate driven by host genetics was related to the improvement of insulin response after the oral glucose tolerance test. The abundance of *Eubacterium* and *Roseburia intestinalis* increased, and the abnormal level of production or absorption of propionate was related to the increased risk of T2DM. Accordingly, butyrate can promote postprandial insulin secretion and propionate generation in feces, which elevates the risk of T2DM. Nevertheless, more prospective cohort studies are warranted in different regions for deep exploration.

## 4. The Function of Gut Microbiota in T2DM Pathophysiology

**Figure 1:**
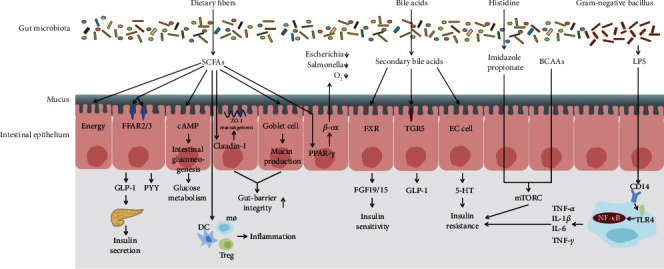
The main mechanisms between gut microbiota and T2DM. SCFAs mediate glucose homeostasis by energy supply for colonocytes, increasing intestinal hormone secretion and gluconeogenesis, decreasing gut permeability, maintaining intestinal anaerobic environment, and regulating host immune. Imidazole propionate and BCAAs can block insulin signaling and activate mTORC1 responsible for insulin resistance. Bile acids have effects on glucose metabolism by binding to FXR and TGR5 and stimulate the release of 5-hydroxy tryptamine in enterochromaffin cells to induce insulin resistance. LPS induces low-grade inflammation and insulin resistance by binding to TLR4.

Metabolites and components of gut microbiota affect the progress of various diseases primarily through distinct signaling pathways (Figure 1). It has been well documented that SCFAs, bile acid, branched-chain amino acids (BCAAs), imidazole propionate, and lipopolysaccharide (LPS) are important regulators in T2DM. We have discussed the detailed mechanism as follows.

### 4.1. SCFAs

SCFA is a metabolite produced by intestinal bacteria to metabolize dietary fiber, including acetate, propionate acid, and butyrate [19]. As one of the most extensively studied metabolites, SCFAs affect glucose metabolism and insulin sensitivity by participating in a variety of pathways, thereby affecting the development of diabetes. Their core functions can be summarized as follows: (1) Stimulating the secretion of intestinal hormones: SCFAs can be used as energy-regulated signaling molecules, directly bound to the free fatty acid receptor (FFAR2 or FFAR3) on the surface of intestinal L cells, and stimulate the secretion of peptide YY (PYY) and glucagon-like peptide-1 (GLP-1) by colon L cells. These two intestinal hormones are responsible for delaying gastric emptying, inhibiting appetite, promoting insulin secretion, and reducing glucagon [20–23]. (2) Energy supply: SCFAs account for 5%-10% of total energy consumption in normal colon, especially butyrate [24]. (3) Increasing the intestinal gluconeogenesis: by acting in a cAMP-dependent mechanism, butyrate is able to promote the expression of intestinal gluconeogenesis-related genes. Besides, propionate is an important substrate of gluconeogenesis, which activates the intestinal gluconeogenesis-related genes through the intestinal-brain nerve circuit including FFAR3 regulating blood glucose and lipid metabolism [25]. (4) Maintaining the integrity of the intestinal barrier: the intestinal barrier in T2DM patients is damaged by proinflammatory components, including LPS resulting in insulin resistance [26, 27]. Butyrate enhances the integrity of intestinal barrier by increasing the expression of Claudin-1 mediated by the interaction between transcription factor SP1 and specific modification in the promoter region of binding protein Claudin-1, which leads to the redistribution of ZO-1 and Occludin on cell membrane [28]. Gut microbiota has been proved to operate host-secreted mucus glycoproteins as a source of nutrients under dietary fiber deficiency, and it can cause the degradation of colonic mucus barrier [29]. The addition of butyrate and propionate to human goblet-like LS174T cells can increase the expression of MUC2 mediated by acetylation/methylation of AP-1 and MUC2 promoter histones. MUC2 synthesis increases intestinal mucus thickness, ultimately leading to a decrease of intestinal permeability and protection of the integrity of intestinal barrier [30]. Moreover, acetate has been implicated as a good regulator in reducing mucosal permeability and enhancing intestinal barrier [31]. (5) Maintenance of intestinal anaerobic environment: butyrate activates peroxisome proliferator-activated receptor g (PPAR-g) in colon cells and drives the energy metabolism of colon cells to transform to *β*-oxidation. In addition, butyrate reduces the bioavailability of respiratory electron receptors of *Enterobacteriaceae* in the colon cavity and prevents the abnormal proliferation of opportunistic pathogen bacteria, *ca. Escherichia coli* and *Salmonella*, and maintains the intestinal microecological balance in T2DM patients [32]. (6) Enhanced immunity: patients with T2DM are associated with chronic low-grade inflammation [33, 34]. Symptoms of patients can be alleviated by inhibiting inflammation. Propionate can change the hematopoietic function of bone marrow in mice and increase the production of macrophages and dendritic cells [35]. Butyrate has an anti-inflammatory effect by promoting the production of regulatory T cells (Treg) and reducing inflammation [36–39].

### 4.2. Bile Acid

The primary bile acids are synthesized from cholesterol in the liver. Primary bile acids are secreted into the intestine and often converted into secondary bile acids by gut microbiota, affecting glucose metabolism and insulin sensitivity through different signaling pathways [40]. Secondary bile acid stimulates the farnesoid X receptor (FXR) and leads to the release of fibroblast growth factor 19/15 (FGF19/15). FGF19/15 acts as ligands to improve insulin sensitivity and glucose tolerance. Secondary bile acid can also activate the thiol guanosine receptor-5 (TGR-5) receptor, promote muscle energy consumption and the secretion of GLP-1 by intestinal L cells, and rescue insulin resistance and abnormal glucose metabolism [41, 42]. Gut microbiota can transform primary bile acids into secondary bile acids, regulate bile acid diversity, and decouple them through bile salt hydrolase, which is essential in bile acid synthesis, modification, and signal transduction. Abnormal bile acid metabolism mediated by gut microbiota will affect the role of bile acid in the regulation of glucose metabolism [43, 44]. Recent studies have shown that the metabolism of bile acid is abnormal in patients with gut microbial dysbiosis with increased secretion of secondary bile acids such as lithocholic acid and deoxycholic acid. Bile acid stimulates the release of 5-hydroxy tryptamine by enterochromaffin cells, resulting in reduced insulin release and enhanced glucagon secretion [45]. Furthermore, bile acid can also directly cause altered structure, function, and stability of intestinal flora [46].

### 4.3. LPS

A number of studies have shown that T2DM patients have a low degree of inflammation due to increased LPS in the peripheral circulation [47–49]. High levels of serum LPS are mainly produced by gram-negative bacteria, which increases the intestinal permeability and leads to elevated LPS in the peripheral circulation. LPS recognizes the receptor TLR4 with the help of CD14, which leads to macrophage aggregation and NF-*κ*B inflammatory signaling pathway activation, characterized by elevated production of inflammatory factors such as TNF-*γ*, IL-1*β*, IL-6, and TNF-*α*. After that, abnormal phosphorylation of insulin receptor substrate and insulin resistance occur [50]. Furthermore, *β* cells are damaged followed by inhibited insulin secretion and downregulated expression of the Homeobox1 (PDX1) gene in the pancreas and duodenum [51, 52].

### 4.4. BCAAs

BCAAs mainly include leucine, isoleucine, and valine, which are essential amino acids for the human body. They cannot be synthesized by the host, must be obtained from diet, and are mainly produced by gut microbiota metabolism [53]. Elevated plasma level of BCAAs is a risk factor for T2DM [54–56]. Increased intake of BCAAs in diet promotes the development of T2DM and insulin resistance [57, 58]. The metagenome of gut microbiota in 277 nondiabetic and 75 diabetic patients has suggested that the functional genes of BCAA synthase and internal transport proteins were enriched in T2DM patients [59]. The main driving bacteria for the synthesis of BCAAs are *Prevotella copri* and *Bacteroides vulgatus*. Feces microbiota transplantation of *P. copri* in a mouse model can cause the insulin resistance, decrease glucose tolerance, and increase plasma BCAAs. The mechanism of insulin resistance induced by BCAA is found to be closely related to the mTOR signaling pathway. High expression of phosphorylated mTOR^Ser2448^, phosphorylated S6K1^Thr389^, and phosphorylated IRS1^Ser302^ has been found in mice fed with BCAAs, which can block the normal conduction of insulin signaling and cause insulin resistance [60]. In addition, BCAAs can increase the oxidation of free fatty acids and activate phosphatidylinositol 3 kinase (PI3K). PI3K activation can further induce insulin resistance through AKT phosphorylation [61]. However, further research is needed in that the exact molecular mechanism remains unclear.

### 4.5. Imidazole Propionate

The study by Koh et al. has elaborated the new mechanism that imidazole propionate, a product of histidine metabolism in the intestinal flora, affected glucose metabolism through the mTORC1 signaling pathway [62]. It has been clarified that imidazole propionate directly exerts effects on p38*γ* mitogen-activated protein kinase (MAPK), promotes p62 protein phosphorylation, and then induces mTORC1^S2448^ phosphorylation. The downstream S6K1 protein phosphorylation is further activated, which causes abnormal serine phosphorylation of insulin receptor substrate, IRS degradation, and insulin resistance. A recent multicenter cohort study has revealed that imidazole propionate increased in prediabetes and T2DM patients with Bacteroides 2 enterotype and low abundance of microbial genes. It is positively associated with the richness of *Clostridium baumannii*, *Clostridium parasymbiotics*, and *Ruminococcus gnavus* and negatively associated with anti-inflammatory bacteria [63]. Imidazole propionate was also positively correlated with systemic inflammation [63].

## 5. Therapies for T2DM Based on Gut Microbiota

In view of the important role of gut microbiota in T2DM pathophysiology, the prevention and treatment of diabetes by regulating intestinal flora is currently a research hotspot. Many studies have found that the characteristics of individual intestinal flora can affect the effect of traditional treatment.

### 5.1. Fecal Microbiota Transplantation

Fecal microbiota transplantation (FMT) is a useful treatment strategy for gastrointestinal diseases with ineffective antibiotic treatment, including ulcerative colitis, *Clostridium difficile* infection, and irritable bowel syndrome [64]. It is very effective in the treatment of *C. difficile* infection [65], and 60% of patients were cured within 1 month without serious adverse consequences [66]. In 2012, the first clinical trial of FMT in the treatment of metabolic syndrome was reported, in which nine patients with metabolic syndrome received fecal microbiota from healthy lean donors [66]. After six weeks of treatment, the insulin sensitivity was significantly improved, and the butyrate-producing bacteria increased [67]. Another larger study has demonstrated the beneficial effect of FMT in the treatment of metabolic syndrome [68]. The fecal microbiota transplantation from healthy donors to patients can effectively improve peripheral insulin resistance in the short term (6 weeks), with a decrease in HbA1c and an increase of plasma *γ*-aminobutyric acid (GABA) level. However, there was individual difference in patients' response to FMT. Patients with low baseline intestinal flora diversity showed a more significant effect, suggesting that the characteristics of patients' gut microbiota were crucial factors affecting the treatment. de Groot et al. have further demonstrated the effect of homologous FMT donors' own metabolic characteristics on FMT by using gastric bypass donor (RYGB-D) and metabolic syndrome donor (METS-D) [69]. The results showed that the insulin sensitivity of patients with metabolic syndrome after 2 weeks of transplantation of METS-D intestinal flora was significantly reduced, accompanied by the increase of lithocholic acid, deoxycholic acid, and isolithocholic acid. However, the intestinal transit time of patients receiving RYGB-D intestinal flora was reduced, and the expression and plasma level of chemokine ligand 2 (CCL2) were increased, implicating that the donor should be carefully selected in the treatment of FMT.

### 5.2. Dietary Fiber

Change of food structure is the most basic auxiliary means for the treatment of diabetes, among which dietary fiber has attracted much attention due to its strong effect on improving T2DM [70, 71]. According to the *Chinese Diabetes Dietary Guidelines 2019*, T2DM patients should increase dietary fiber intake by 25-30 g/d, and whole grains and legume carbohydrates should account for 1/3 of the staple food. A large-scale long-term study from three forthcoming cohorts has found that the increasing amount of total whole-grain intake and commonly consumed whole-grain foods results in a decreased risk of type 2 diabetes, providing strong support for daily food recommendations of sufficient whole-grain consumption as a health-giving diet style to prevent type 2 diabetes [72]. Ma-pi diet is rich in complex carbohydrates, beans, fermented products, sea salt, and green tea, which can increase the diversity of gut microbiota, enrich short-chain fatty acid-producing bacteria and mucus-producing bacteria, such as *Faecalibacterium*, *Akkermansia*, *Lachnospira*, *Bacterioides*, and *Roseburia*, inhibit *Collinsella* and *Streptococcus* bacteria with proinflammatory effect, and reduce inflammation in T2DM [73, 74]. WTP diet is a high dietary fiber diet based on whole grain, supplemented by traditional Chinese medicine homologous food and probiotics [75]. The dietary fiber can enrich SCFA-producing bacteria, improve the level of intestinal SCFAs, prevent the growth of harmful bacteria, activate intestinal cells to secrete GLP-1, and increase insulin and HbA1c levels in patients. The abundance of SCFA-producing bacteria is higher with reduced HbA1c. It is worth noting that gut microbiota will also affect the response of individuals to dietary fiber. The analysis of gut microbiota between individuals with well or poor glucose metabolism after eating barley bread has revealed that *Prevotella* exists in most individuals who respond to barley bread compared with those without response to dietary intervention [76]. *Prevotella* is considered to be directly related to the effect of high fiber intake.

### 5.3. Probiotics

Probiotics, generally gram-positive bacteria, are defined as live microorganisms which confer health benefits on human health at an adequate level. According to current research, there are mainly three types of probiotics for diabetes: (1) common probiotics. These types of bacteria mainly from *Lactobacillus* and *Bifidobacterium* are widely used in food fermentation and have high safety in use [77, 78]. The intervention of probiotics in T2DM patients has been carried out in many clinical trials, but the results are inconsistent. Meta-analysis of multiple clinical trials showed that probiotics could effectively reduce FBG, fasting insulin, and HbA1c and improve the efficacy of HOMA-IR [79]. Another meta-analysis found that probiotics effectively reduced oxidative stress markers (TAS, TAS and MDA), and the benefits in reducing HbA1c were not clear, suggesting that probiotics mainly alleviated diabetes by improving oxidative stress rather than glucose metabolism [80]. Sun et al. meta-analysis showed that multistrains combined with probiotic capsules were able to effectively reduce FBG and HbA1c in patients with T2DM, but the effect was not significant in patients with other risk factors [81]. The inconsistent results might be attributed to the use of strains, doses, intervention time, and single or multiple strains; thus, more clinical trials are needed to evaluate the role of conventional probiotics in preventing and improving T2DM [82]. (2) Novel probiotics. *A. muciniphila* has recently become the new candidate for probiotics to improve a variety of diseases [83–85]. The abundance of *A. muciniphila* in T2DM patients is significantly reduced [86]. Oral administration of *A. muciniphila* in mice can improve the secretion of GLP-1 in mouse colon cells [87], improve glucose tolerance [88], restore intestinal barrier, and reduce inflammation [89]. In addition, the application of *A. muciniphila* pasteurization agent and specific membrane proteins isolated from *A. muciniphila* in obese mice with diabetes significantly reduces the fat content in mice and improves insulin resistance and dyslipidemia [90]. Depommier et al. have reported *A. muciniphila* in obese patients with insulin resistance [91]. Supplementation of pasteurized *A. muciniphila* significantly improved insulin resistance and lipid metabolism but reduced liver damage and inflammation-related plasma markers. Another potential probiotic for T2DM is *Faecalibacterium prausnitzii*, an important butyrate-producing bacterium, which is negatively correlated with T2DM [13]. In db/db mice, its anti-inflammatory metabolite AMA restores the structure and function of the intestinal barrier by regulating the tight junction pathway and the expression of ZO-1 [92]. In addition, *F. prausnitzii* was significantly enriched in the treatment of diabetes through diet, fecal transplantation, drugs, and other measures (described below), which suggested that *F. prausnitzii* was very important in the treatment of T2DM. However, the application safety of this bacterium is still not clear due to a lack of effective human research. (3) Genetic engineering bacteria. The strain was genetically modified to produce biological factors for the disease. One example related to T2DM is the application of *Lactococcus lactis* as a carrier to produce GLP-1 [93]. Oral administration of the *Lactococcus lactis* can reduce blood glucose and increase insulin concentrations in rats. Nevertheless, there is still a long way to go for clinical applications of probiotics in T2DM in the future.

### 5.4. Exercise

Exercise is essential for improving insulin sensitivity, reducing blood glucose, and inhibiting inflammation [94, 95]. According to the *Guideline for prevention and treatment of type 2 diabetes in China*, adult patients with T2DM walk fast at least 150 min per week, play Taijiquan, cycling, table tennis, and golf, and exercise muscle strength and endurance by resistance exercise 2-3 times per week. The study of gut microbiota provides a new perspective to the mechanism by which exercise regulating diabetes. Exercise not only increases the diversity of gut microbiota but also increases the abundance of *A. muciniphila* in athletes' intestinal flora, which is negatively correlated with obesity and T2DM [96]. Accumulated studies have shown that the abundance of *Bacteroides* increased after exercise training, while the abundance of *Clostridium genus* and *Blautia* decreased [97]. The change of gut microbiota has positive correlation with the absorption of glucose, implicating that exercise decreased the blood glucose level of diabetic patients through gut microbiota. Gut microbiota can regulate diabetes through the SCFA/FFAR/GLP-1 signaling pathway [98]. The antidiabetic efficacy of exercise is also affected by individual gut microbiota. Studies on drug-naive and overweight diabetic patients showed that the glucose homeostasis and insulin sensitivity regulated by exercise have significant relationship to the feature of gut microbiota and the ability to metabolize and ferment proteins and carbohydrates [99]. The gut microbiota of the responders who benefit from exercise has enhanced the ability to synthesize SCFAs, GABA, and decompose BCAAs, while the gut microbiota of the nonresponders has produced more harmful metabolites. The fecal microbiota transplantation in mice has confirmed that gut microbiota improved insulin resistance by exercise. The baseline gut microbiota characteristics of patients, such as the abundance of *Bacteroides* and concentration of GABA, can accurately predict the response to exercise intervention.

## 6. Gut Microbiota and Antidiabetic Drugs

### 6.1. Metformin

Since the inception of metformin in the mid-20th century, it has become the first choice for the treatment of T2DM, which can inhibit liver gluconeogenesis and improve insulin sensitivity. However, the specific mechanism of action of metformin is still under exploration. Numbers of studies have shown that the hypoglycemic mechanism of metformin may be mediated by gut microbiota. To the best of our knowledge, metformin affects glucose metabolism targeting gut microbiota in the following aspects: (1) Balancing the ecology of intestinal microorganism, improving the disorder of gut microbiota in T2DM patients, and making it closer to healthy individuals [9]. (2) Improving the synthesis ability of SCFAs, increasing the abundance of SCFA-producing bacteria, such as *Shewanella*, *Lactobacillus*, *Blautia Bifidobacterium*, *Akkermansia*, *Prevotella*, *Megasphaera*, and *Butyrivibrio* species [9, 11, 100]. As mentioned above, SCFAs can improve insulin sensitivity and exert a hypoglycemic effect by increasing the secretion of GLP-1 and PYY. Studies have shown that metformin can indeed improve the plasma and intestinal GLP-1 levels in T2DM patients [101–103]. (3) Regulating the glucose metabolism through the bile acid pathway. Sun et al. elaborated that metformin played a hypoglycemic effect through intestinal *Bacteroides fragilis*-bile acid GUDCA-intestinal FXR metabolic axis [10]. Metformin inhibited the growth of *B. fragilis* by modifying the metabolism of folic acid and methionine, thereby reducing the activity of bile acid hydrolase BSH and increasing the level of GUDCA. As an endogenous competitive antagonist of intestinal FXR receptor, GUDCA inhibited the FXR signaling pathway. Inhibition of the intestinal FXR signaling pathway could increase the secretion of GPL-1 and regulate glucose metabolism [104]. (4) Increasing the abundance of probiotics. In the study of gut microbiota, the increase in the abundance of probiotics (such as *Lactobacillus* and *Bifidobacterium*) after metformin administration is common [8, 10, 100]. A recent study on mice showed that SGLT1 activated in the proximal intestine of mice through glucose induction could reduce glucose production. A high-fat diet reduced the expression of SGLT1 in the proximal ileum of mice and reduced the abundance of *Lactobacillus*. Metformin offsets these microbial changes and restores glucose sensing [105], but whether this phenomenon exists in humans needs further study. (5) Effected by imidazole propionate. As described above, imidazole propionate blocks insulin conduction through the mTORC1 signaling pathway and then induces diabetes. Recent studies showed that imidazole propionate also determines the hypoglycemic effect of metformin [106]. In T2DM patients who take metformin but still have high blood glucose, the concentration of imidazole propionate is higher. The mouse experiment confirmed that imidazole propionate destroyed the hypoglycemic effect of metformin. Further cytological experiments revealed that imidazole propionate inhibited metformin-induced adenosine 5′-monophosphate-activated protein kinase (AMPK) activation by inhibiting AMPK phosphorylation through the p38*γ*/Akt pathway. The p38*γ* kinase inhibitor could effectively block the inhibitory effect of imidazole propionate on metformin, which provided a new idea for the individualized treatment of diabetic patients. In addition, up to a third of patients who take metformin were reported to have gastrointestinal side effects, such as diarrhea, abdominal distension, and nausea. It has been demonstrated that metformin significantly increased the abundance of *Escherichia coli* and upregulated the corresponding virulence factors and gas metabolism genes [9, 11].

### 6.2. Acarbose

Acarbose is an *α*-glucosidase inhibitor, which reduces the digestion and absorption of carbohydrates in the small intestine, thereby reducing the postprandial blood glucose level. Acarbose completely acts in the intestine, which may partially affect the composition of distal gut microbiota. A previous study has shown that the gut microbiota of prediabetes who took acarbose changed significantly, where the abundance of *Lactobacillus*, *Faecalibacterium*, and *Dialister* was enriched [107]. Among them, *Dialister* was negatively correlated with HbA1c in prediabetes patients, indicating its potential role in glucose metabolism regulation [107]. Acarbose can also increase the abundance of *Bifidobacterium longum* and *Enterococcus faecalis* in patients with T2DM. *E. faecalis* were negatively correlated with LPS concentration, while *B. longum* was positively correlated with HDL cholesterol concentration [108]. The data from newly diagnosed T2DM patients showed that acarbose increased the abundance of probiotics *Lactobacillus* and *Bifidobacterium*, while the content of *Bacteroides* was significantly reduced. The metabolic spectrum of bile acid in plasma and feces has changed, suggesting that acarbose may affect the gut-mediated bile acid signal pathway and improve the glucose metabolism [109]. In addition, similar to metformin, the baseline characteristics of individual gut microbiota determine the therapeutic effect of acarbose. The metabolic parameters of patients with intestinal flora dominated by *Bacteroides*, including FBG, insulin, C-peptide levels, and insulin resistance, are improved more significantly compared with patients with intestinal flora dominated by *Prevotella* [109]. Accordingly, stratification of T2DM patients according to gut microbiota characteristics before treatment is useful for individualized treatment.

### 6.3. Traditional Chinese Medicine

Traditional Chinese medicine has shown some advantages in improving the life quality of T2DM patients by influencing insulin resistance [110, 111]. Therefore, the *Guideline for prevention and treatment of type 2 diabetes in China* takes traditional Chinese medicine as an adjuvant drug for T2DM treatment. Many components of traditional Chinese medicine do not enter the blood, but it has a clear effect. Increasing data has suggested that its efficacy may be mediated by gut microbiota [112]. Gegenqinlian decoction can significantly enrich the abundance of *F. prausnitzii* in the intestinal tract of T2DM patients, which has significantly negative correlation with FBG, HbA1c, and postprandial 2 h blood glucose levels and has positive correlation with HOMA-*β* [113]. AMC, an herbal formula containing eight traditional Chinese medicines, can effectively alleviate hyperglycemia and hyperlipidemia in diabetic patients and alter the gut microbiota of T2DM patients [114]. They significantly increase the symbiotic bacteria utilized by *Blautia* spp., which is associated with lipid homeostasis and glucose. AMC shows superior effects in improving insulin resistance (HOMA-IR) and plasma triglycerides and plays a vital role in regulating gut microbiota, and only AMC increases the symbiotic bacteria using *Faecalibacterium* spp. It has been shown that berberine can effectively reduce the HbA1c of patients, and the effect of berberine in inhibiting the biotransformation of dicarboxylic acid is mediated by *Ruminococcus bromii* in T2DM [115].

## 7. Conclusion

Gut microbiota plays an important role in T2DM by exerting effects both in composition and function. A decrease of butyrate-producing bacteria, such as *Faecalibacterium* and *Roseburia*, and reduction of butyrate are common in T2DM, which may be the principal causes of T2DM. Several factors associated with gut microbiota have been elucidated in T2DM, including SCFAs, bile acids, LPS, BCAAs, and imidazole propionate. Gut microbiota can not only be used as a diagnostic biomarker but a potential therapeutic target for T2DM. Nevertheless, characteristics of individual gut microbiota have an important influence on different treatments, particularly the ratio of *Prevotella/Bacteroides*. As a result, the treated subjects can be divided into responders and nonresponders (Table 1). However, the exact core driving bacteria or flora is still unclear. Therefore, multicenter and comprehensive studies are warranted for further investigation. Additionally, the multiomics has been extensively applied for gut microbiota research, such as metagenomics, transcriptomics, proteomics, and metabolomics exploring the role of gut microbiota in T2DM. Elucidation of the precise role and mechanism of gut microbiota in T2DM will provide novel insight into developing individualized therapy for T2DM patients.

## Figures and Tables

**Table 1:** Baseline gut microbiota or bacterial metabolite characteristics of responders and nonresponders.

Treatment	Responders		Nonresponders		Predictors
	Increase	Decrease	Increase	Decrease	
Exercise	*Lanchospiraceae bacterium* *Streptococcus mitis* *Bacteroides* SCFAs GABA	*Bacteroides xylanisolvens* *Alistipes shahii* *Prevotella copri* BCAAs	*Alistipes shahii* Detrimental metabolites	*Alistipes putredinis* *Ruminococcus gnavus* GABA SCFAs	*Bacteroides xylanisolvens* *Bacteroides cellulosilyticus* GABA

FMT	*Subdoligranulum variabile* *Dorea longicatena*	*Eubacterium ventriosum* *Ruminococcus torques*	*Ruminococcus torques*		Low baseline diversity *Subdoligranulum variabile*

Barley bread	*Prevotella* species		*Bacteroides* species		*Prevotella copri*

Drugs					

Metformin		Imidazole propionate	Imidazole propionate		Imidazole propionate

Acarbose	*Bacteroides* UDCA PBA/SBA ratio	LCA and DCA 12-*α* OH/non-12*α* OH BA ratio	*Prevotella*		*Bacteroides*/*Prevotella*

SCFAs: short-chain fatty acids; GABA: *γ*-aminobutyric acid; BCAAs: branched-chain amino acids; FMT: fecal microbiota transplantation; UDCA: ursodeoxycholic acid; PBA: primary bile acids; SBA: secondary bile acids; LCA: lithocholic acid; DCA: deoxycholic acid; BA: bile acid.

## Data Availability

No data were used to support this study.
